# Adaptation to sequence force perturbation during vertical and horizontal reaching movement—averaging the past or predicting the future?

**DOI:** 10.3389/fnsys.2012.00060

**Published:** 2012-08-14

**Authors:** Firas Mawase, Amir Karniel

**Affiliations:** Department of Biomedical Engineering, Ben-Gurion University of the NegevBeer-Sheva, Israel

**Keywords:** motor memory, vertical reaching, force perturbations, grasping, reaching movements

## Abstract

Several studies conducted during the past decade have suggested that episodic memory is better equipped to handle the future than the past. Here, we consider this premise in the context of motor memory. State-of-the-art computational models for trial-by-trial motor adaptation to constant and stochastic force field perturbations in a horizontal reaching paradigm have shown that motor memory registers a weighted sum of past experiences to predict force perturbation in a subsequent trial. In the current study, we used the standard horizontal reaching movement paradigm and a novel vertical reaching movement paradigm to test motor memory function during adaptation to force fields increasing in magnitude in a simple predictable linear series. We found that adaptation to constant and sequence force fields are similar in vertical and horizontal reaching. For both horizontal and vertical reaching, we found that the expectation in a particular trial was the average of the previous few trials rather than an expectation of a larger perturbation, as would be expected from a simple extrapolation. These findings are not consistent with those of our previous studies on lifting and grasping tasks, in which we found that the grip force is correctly adjusted to the next weight in a series of tasks with gradually increasing weights, thus predicting the future rather than averaging the past. The results of the current study devoted to reaching movements and of our previous study addressing a lifting task suggest that the brain can generate at least two different types of motor representation, either addressing the past in reaching or predicting the future in lifting. We propose that prior experience and the effect of environment's variability are the reasons for the observed differences in expectation during lifting and reaching. Finally, we discuss these two types of memory mechanisms with respect to the distinct neural circuits responsible for lifting and reaching.

## Introduction

Episodic memory is classically regarded as a neuronal cognitive system that allows past experience to be remembered, namely, that enables people to recall the content and timing of incidents in specific past events (Tulving, 2002). However, many recent cognitive studies have recognized the close relationship between remembering the past and imagining the future (Dudai and Carruthers, 2005; Gilbert and Wilson, 2007; Schacter and Addis, 2007a,b; Addis et al., 2009, 2011; Dudai, 2009). Even though the dichotomy between handling the past and the imagining future has been studied almost exclusively in relation to episodic memory, there are a few clear similarities between motor memory and episodic memory that justify the consideration of this dichotomy for motor memory as well. For both types of memory, there is strong evidence for involvement of the hippocampus: it has, for example, been observed that the hippocampus and the striatum interact during motor sequence consolidation (Albouy et al., 2008). In addition, it has been proposed that the hippocampus forms part of a common core brain network during remembering the past and imaging the future by using episodic memory (Addis and Schacter, 2008; Addis et al., 2011). Moreover, two main features of memory, consolidation, and mental practice, have been studied in the context of motor control (Brashers-Krug et al., 1996; Eisenberg and Dudai, 2004; Krakauer and Shadmehr, 2006). However, the possible relationship between motor memory and past–future issues remains almost entirely unexplored and it is this relationship that we address in the current study.

People demonstrate excellent motor ability when learning a new environment. Numerous studies have examined the processes involved in motor adaptation during reaching movements when the study subjects are exposed to different novel environments, such as visuomotor perturbations (Flanagan and Rao, 1995; Wolpert et al., 1995; Krakauer et al., 2000; Mazzoni and Krakauer, 2006), mechanical perturbations (Shadmehr and Mussa-Ivaldi, 1994; Shadmehr and Brashers-Krug, 1997) or time-dependent perturbations (Levy et al., 2010). Some of these studies focused on the effect of prior statistics of the environment on motor adaptation (Scheidt et al., 2001), while others investigated motor memory retention (Huang and Shadmehr, 2009) and inter-limb transfer (Criscimagna-Hemminger et al., 2003; Malfait and Ostry, 2004). Current theories and computational models about motor adaptation in these force-field paradigms suggest that the human brain predicts a future external perturbation on the basis of a weighted sum of past experience (Thoroughman and Shadmehr, 2000; Scheidt et al., 2001; Takahashi et al., 2001; Donchin et al., 2003). However, the process of adaptation for a series of gradually increasing force perturbations in reaching movements is still not fully understood. What happens when we experience trial-by-trial series of 1N, 2N, 3N? Do we expect the average 2N or predict the next 4N? How does the movement's direction affect this prediction? In this study we tried to answer these open questions.

Here, we tested short-term motor memory during vertical and horizontal reaching movements under conditions of a trial-by-trial sequence of gradually increasing velocity-dependent force perturbations and sought to determine whether the subjects exposed to this series of force fields would base their predictive control on the past trials (i.e., the average of past perturbations) or whether they would demonstrate the ability to anticipate the future (i.e., the next perturbation).

## Materials and methods

### Subjects

**Figure 1:**
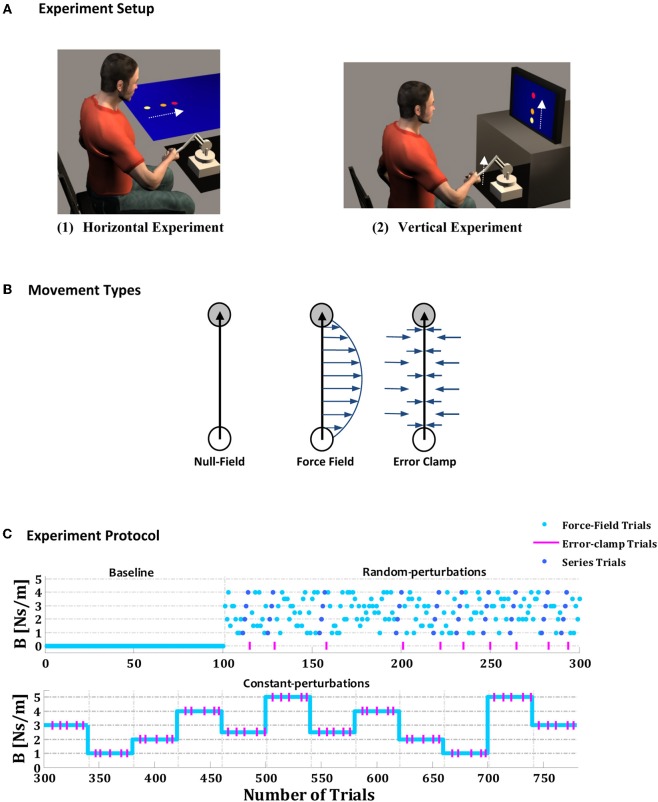
**Experimental setup, movement types and experimental protocol**. **(A)** Subjects were instructed to make a point-to-point reaching movement in the horizontal (1) or vertical (2) plane while holding the handle of a two-degrees of freedom robotic manipulandum. **(B)** Three types of trial were performed: null trials, force-field trials, and error-clamp trials. During the null-field trials, the robot's motors were turned off, and no force field was applied. During the force-field trials, the robot's motors were used to produce a viscous force field (arrows) on the subject's hand proportional in magnitude and perpendicular in direction to the instantaneous the velocity of hand motion. During the error-clamp trials, the lateral error was clamped between the initial hand position and the center of the target by using “force channel,” which effectively counteracted the lateral motion and forced nearly straight line movements to the target. **(C)** Experimental protocol, which comprises three phases: a baseline phase (100 trials), a random-perturbations phase that included 10 embedded series of force fields with increasing amplitudes (200 trials), and a constant-perturbations phase that included 12 blocks of constant force fields (40 trials in each block, to give a total of 480).

Twenty naive self-proclaimed right-handed subjects (11 men, 9 women, mean age 24.3 years) participated in this study. All subjects signed an informed consent form as required by the local Helsinki Committee. Subjects were randomly assigned to one of two groups with identical experimental protocols; one group (*n* = 10, 4 men and 6 women) performed horizontal reaching movements (experiment 1, Figure 1A1), and the other group (*n* = 10, 7 men and 3 women) performed vertical reaching movements (experiment 2, Figure 1A2).

### General task description

Subjects were instructed to make a point-to-point reaching movement in the horizontal plane (for experiment 1) or in the vertical plane (for experiment 2) while holding the handle of a haptic robotic manipulandum (Phantom 1.5TM by SensAble), with two degrees of freedom, that applied forces to the hand. The robot measured hand force, position, and velocity, at a sampling rate of 200 Hz. Visual information was provided to the subjects through horizontally and vertically oriented screens in the horizontal and vertical experiments, respectively. Two circles (7 mm in diameter) indicated the locations of the initial point (white circle) and the target point (red circle) toward which the subject was instructed to make point-to-point reaching arm movements. An on-screen cursor comprising a small circle (5 mm diameter) tracked the subject's movements (Figure 1A). The subject was instructed to reach the target as quickly as possible. For each trial, the computer provided visual feedback on the duration of the movement to reach the target as follows: whether the duration of the movement was appropriate (movement duration 0.2–0.7 s), too slow (movement duration > 0.7 s), or too fast (movement duration < 0.2 s).

The methods applied in the current study were similar to those used in previous studies on hand motor control (Scheidt et al., 2000, 2001; Joiner and Smith, 2008; Joiner et al., 2011). Three types of trial were used—null, force-field, and error-clamp (Figure 1B). During null-field trials, no external force field was applied, and the hand of the subject moved in free space. During force-field trials, the motors of the robot were used to produce an external viscous force field on the subject's hand, which was proportional in magnitude and perpendicular in direction to the speed of the hand (*f* = *Bv*, where *f* represents the force applied by the robot, *v* the subject's hand speed, and *B* the amplitude of perturbation). In the error-clamp trials, the displacement lateral error was clamped between the initial hand position and the center of the target by using a virtual haptic “force channel,” which effectively counteracted lateral motion and forced approximately straight-line hand movements. The force channel was implemented as a stiff, viscous one-dimensional spring, with 1000 N/m, and a damper, of 50 Ns/m, in the axis perpendicular to the target direction (Scheidt et al., 2000; Smith et al., 2006; Joiner and Smith, 2008; Joiner et al., 2011). The error-clamp trials were used for high-accuracy measurement of the adaptation level during the learning of the new environment; note that the method involves only minimal after effects due to the minimal spatial errors. Movements were always made along the positive y direction, and perturbing forces were always directed to the right (Figure 1B).

Each experiment, comprising a total of 780 trials, was divided into three phases: baseline, random-perturbations and constant-perturbations (Figure 1C). The first phase consisted of a baseline phase comprising 100 trials, during which no force field was applied. The second phase comprised the random perturbations phase of 200 trials, during which the subjects performed movements in a random viscous force field. In this phase, the forces applied to the subject's hand during the *n*th movement were defined as:

(1) $$\left(\begin{array}{c} F_{X}\\ F_{Y} \end{array}\right)\;=B_{n}\;\cdot \;\left(\begin{array}{cc} 0 & 1\\ 0 & 0 \end{array}\right)\left(\begin{array}{c} \dot{x}\\ \dot{y} \end{array}\right),\;B_{n}\;\in \;\left\{1.0,\;1.5,\;2.0,\;2.5,\;3.0,\;3.5,\;4.0\right\}\;Ns/m$$

where $\dot{x}$ and $\dot{y}$ are the two components of the hand velocity along the lateral (*x*) and distal (*y*) directions, respectively, and *B*_*n*_ is a pseudo-random real number between 1 and 4 Ns/m such that the amplitude of the perturbation force field varied randomly from trial to trial (*B*_*n*_ was distributed uniformly in this phase of the experiment). During the random-perturbations phase it were randomly embedded 10 short linear sequences of force fields, with increasing amplitudes of 1, 2, 3, 4 Ns/m, and continued with error-clamped trials (10% of trials in this phase). The reason for using a random background of forces in this phase was to ensure that information about the series was provided implicitly. In the final—the constant-perturbations—phase, the subject performed 12 blocks of 40 trials in a constant viscous force field. In each block of that third phase, five randomly distributed error-clamps trials were introduced (12.5% of trials in this phase). The blocks were selected randomly but presented in the same order for each subject; the magnitudes of the force fields in each block are shown in Figure 1C. The subjects were not given any information about the types of movement or the phases of the experiment.

### Performance measure and force analysis

We report the displacement at the maximum speed as a measure of movement errors. Our analysis is based on measuring the profiles of the lateral perpendicular forces generated by the subjects during movement in the error-clamped trails. During an error-clamp trial, the measured lateral force reflects an adaptive compensation of the expected lateral force. We examined the feed-forward predictive adaptive compensation of the force-field environment in the random and the constant-perturbations phases of the experiment. During the error-clamp trials, movement errors were very small (average absolute deviations, <1.26 mm in the horizontal experiment and <1.30 mm in the vertical experiment). In the past, researchers seeking to track the expected force field measured either the force that the subjects applied at maximum speed (Wagner and Smith, 2008) or the peak force (Criscimagna-Hemminger et al., 2010) in each force-clamp trial. Since the external force perturbations in our study were velocity dependent, the peak force point will theoretically occur at the peak speed point. We therefore chose the force at maximum speed as the point of our measurements. To ensure that our force results were not sensitive to this specific force measure, we also examined the data for the peak force during the force-channel trials in both experiments. The results were similar for the two cases, and we are therefore confident that our measure of adaptation is robust and independent of force point.

To fully compensate for the external force perturbations in a specific movement, the subjects in our study needed to apply a contrary lateral force (“perfect force”) proportional to the hand velocity on that same trial. The adaptation level was quantified in terms of the correlation coefficient and the adaptation coefficient between the exerted force and the “perfect” force during the error-clamp trials. The correlation coefficient was calculated by a Pearson correlation between the exerted force and the “perfect” force. The value of these coefficients was 1 if the exerted force was identical to the perfect force and 0 if the coefficients were not related. The adaptation coefficient was calculated by linear regression of the exerted force into the “perfect” force (Hwang et al., 2006; Smith et al., 2006). The adaptation coefficient was thus derived from the linear regression between actual and full compensatory force profiles as follows:

(2) $$\begin{array}{l} F_{FC}^{n}=B^{n}\;\cdot \;v_{y}^{n}\\ F_{Actual}^{n}=F_{Error-Clamp}^{n}\\ F_{Actual}^{n}=a\;\cdot \;F_{FC}^{n}\Rightarrow F_{Actual}^{n}=a\;\cdot \;B^{n}\;\cdot \;v_{y}^{n} \end{array}$$

where *F*^*n*^_*FC*_, the full compensatory force (“perfect”) in trial *n*, was calculated as the product of the force field amplitude *B*^*n*^ that was exerted by the manipulandum in trial *n* and the movement velocity in that trial *v*^*n*^_*y*_; *F*^*n*^_*Actual*_ is the measured lateral force in the error-clamp trial *n*; and *a* is the adaptation coefficient.

The database of force-field learning in which the force field was kept constant over 40 trials and was varied between six different force fields (6 force fields × 40 trials × 2 blocks) enables us to compare the learned force field in each block of the constant-perturbations phase with the actual lateral forces in the second phase (when the subjects were exposed to series of force fields).

### Statistical analysis

Statistical analysis of the data was performed using the Matlab software with the Statistics Toolbox (The MathWorks, Natick, MA). *T*-tests were used to compare the force profile between trials. A repeated measure ANOVA was used to compare group effects. The performance of the models was quantified by measuring the correlation and the variance measurement (VAF) between the data and the model's prediction. The significance level was set at 0.05.

## Results

### Adaptation to constant force fields is similar in vertical and horizontal reaching

**Figure 2:**
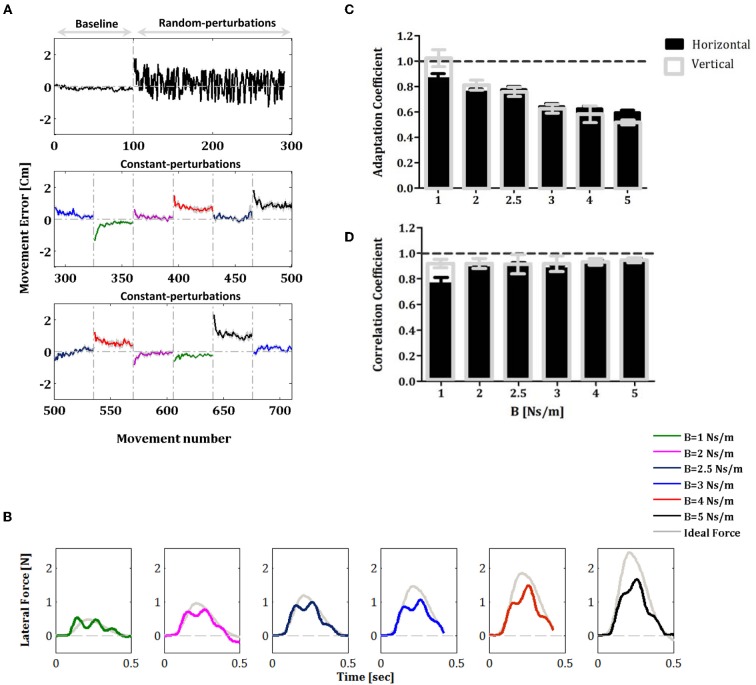
**Movement errors and learning performance of force profiles during adaptation to constant force fields**. **(A)** Movement error averaged across all subjects during the baseline, random-adaptation and constant-adaptation phases. Note that error-clamp trials are not shown in the panels of Figure 2A. The colored traces represent the movement errors that were measured in each condition of force perturbation during the constant-perturbations phase. Errors bars represent SE. **(B)** Lateral force profiles during the constant-perturbations phase. The colored traces represent the lateral force profile averaged across all subjects across the ten error-clamp trials for each force-field condition (1, 2, 2.5, 3, 4, and 5 Ns/m). The gray trace in each panel is the corresponding perfect force profile that is based on the average movement velocity profile that allows full compensation of the force-field perturbation. **(C)** Comparison of the adaptation coefficients for the different force-field conditions during the constant-perturbations phase; black filled bars for the horizontal experiment and gray unfilled bars for the vertical experiment. **(D)** Correlation coefficients for the data presented in B. Error bars represent SE.

All subjects (in the vertical and horizontal experiments) did indeed learn the constant force fields over the constant-perturbations phase, and these results are consistent with previously reported studies (Shadmehr and Mussa-Ivaldi, 1994; Smith and Shadmehr, 2005; Wagner and Smith, 2008). When constant force fields were applied initially in each block of the constant-perturbations phase, the trajectories were distorted in comparison with the trajectories in the null trials of the baseline phase and yielded increasing movement errors. However, as the subjects gained expertise with practice, movement errors in the force fields trials fell and hand trajectories converged to a path very similar to that observed in free space (Figure 2A). As a measurement of adaptation to the new velocity-dependent dynamics, we measured the lateral force field in five error-clamp trials that were randomly interspersed in each block (frequency of one in eight trials, ten error-clamp trials for each condition).

The lateral force profiles measured during the constant-perturbations phase in the horizontal experiment are shown in Figure 2B. The colored traces represent the lateral force profile averaged across all subjects across the ten error-clamp trials for each of the force-field conditions (i.e., 1, 2, 2.5, 3, 4, and 5 Ns/m). The gray trace in each panel represents the corresponding perfect force profile that was based on the average movement velocity profile and the force field magnitude in each block. Note that we obtained similar results in the vertical reaching experiment. To quantify the motor adaptation level for each force-field condition, we calculated the adaptation coefficient. The results presented in Figure 2C show clearly that the subjects adapted to the external forces for all force-field conditions: the adaptation coefficient was significantly different from zero (one sample *t*-test: *P* < 0.001 for horizontal and vertical experiments) and ranged between 0.60 and 0.86 (*r* = 0.86 ± 0.04 (SE) for the first condition, *B* = 1 Ns/m, and *r* = 0.60 ± 0.02 (SE) for the higher perturbation, *B* = 5 Ns/m) for horizontal reaching (black filled bars) and between 0.53 and 1.03 (*r* = 1.03 ± 0.06 for the first condition, *B* = 1 Ns/m, and *r* = 0.53 ± 0.02 for the higher perturbation, *B* = 5 Ns/m) in the vertical reaching experiment (gray unfilled bars). We also calculated the correlation coefficient between the perfect and the actual force profiles (Figure 2D) with the aim to assess matching between forces. We found that for all force-field conditions, the correlations were high (one sample *t*-test: *P*<0.001 for horizontal and vertical experiments) and ranged between 0.76, and 0.94 (*r* = 0.76 ± 0.05 for the first condition, *B* = 1 Ns/m, and *r* = 0.94 ± 0.02 for the higher perturbation, *B* = 5 Ns/m) in horizontal reaching (black filled bars) and between 0.91 and 0.94 (*r* = 0.91 ± 0.03 for the first condition, *B* = 1 Ns/m, and *r* = 0.94 ± 0.02 for the higher perturbation, *B* = 5 Ns/m) in the vertical reaching experiment (gray unfilled bars). Since the force field perturbations were velocity dependent (see section “Materials and Methods”), it was important to determine whether movement velocities were comparable for the various values of *B* (perturbation amplitude) and during the two experiments. We found that the average peak velocity during the different phases of the two experiments were significantly similar and ranged between 53.1–57.8 cm/s and 53.1–55.2 cm/s for the horizontal and the vertical experiments, respectively (one-way ANOVA test: *F*_(6, 63)_ = 0.4562, *P* = 0.84 and *F*_(6, 63)_ = 0.031, *P* = 0.99 for the horizontal and vertical experiments, respectively). We also found that there was no significant difference between the average speed during the trials (one-way ANOVA test: *F*_(6, 63)_ = 0.2419, *P* = 0.96 and *F*_(6, 63)_ = 0.2917, *P* = 0.94 for the horizontal and vertical experiments, respectively). This finding confirms that our measures of adaptation in different phases of the experiments are indeed robust.

### Subjects' predictions were based on the average of the sequence of force fields

**Figure 3:**
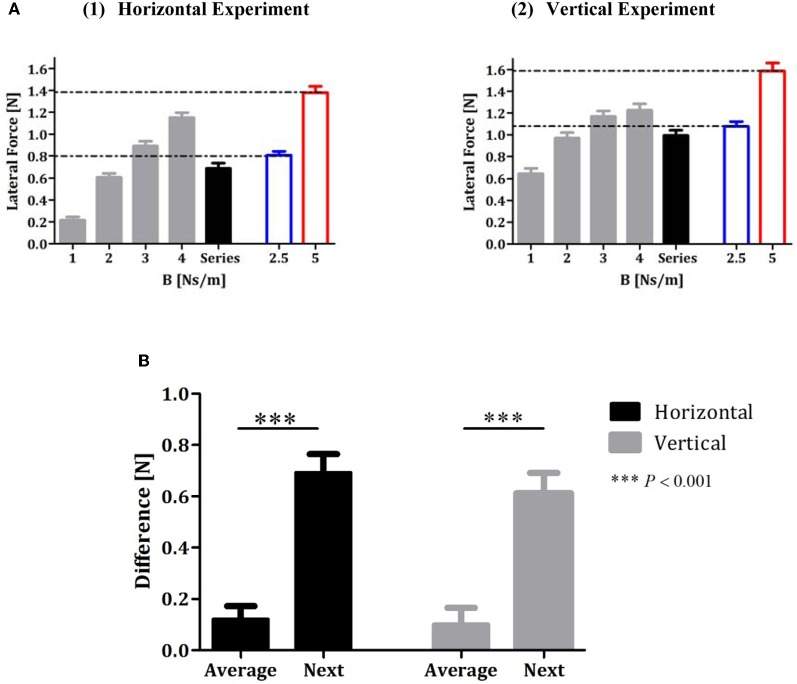
**Analysis of force profiles during adaptation to a series of force-fields**. **(A1** and **A2)** Lateral forces at maximum speed averaged across all subjects for horizontal and vertical experiments, respectively. The gray bars represent the forces during each condition of the constant-perturbations phase (1, 2, 3, and 4 Ns/m), and the black bar represents the lateral forces in the error-clamp trials that followed the series trials in the random-perturbations phase. The blue and red bars represent the lateral forces that subjects applied during constant perturbations of 2.5 and 5 Ns/m, respectively. Error bars represent SE. **(B)** Difference between forces in the error-clamp trials that followed the series trials and forces in two different conditions of constant force-field (“average”- *B* = 2.5 Ns/m and “next”- *B* = 5 Ns/m; black bars for the horizontal and gray bars for the vertical experiments).

An analysis of the lateral force profile in the error-clamp trials that followed the sequence trials indicated that subjects predict approximately the average value of the recent force fields (Figure 3). The gray bars in Figure 3 represent the peak of the lateral force profile averaged across all subjects across the 10 error-clamp trials for each force-fields condition (1, 2, 3, and 4 Ns/m, respectively) in the constant-perturbations phase of the experiment. The black bar represents the peak of the lateral force averaged across all subjects across the ten error-clamp trials that followed the series force-field trials in the random-perturbations phase (Figures 3A1 and A2 for the horizontal and vertical experiments, respectively). For the horizontal experiment, the peak value of the actual force that subjects applied after they had been exposed to a series of force-fields (LF = 0.69 ± 0.05 (SE) [N]) was similar to the peak value of the actual force when they were exposed to the force-field of *B* = 2.5 Ns/m (mean value of the sequence, LF = 0.81 ± 0.04 [N], blur bar in Figure 3A1). This value differed significantly from the learned force when the subjects were exposed to the force-field of *B* = 4 Ns/m (the last term of the series, LF = 1.15 ± 0.04 [N]) or *B* = 5 Ns/m (the next term of the series, LF = 1.38 ± 0.06 [N]). Similar results were obtained in the vertical reaching experiment (Figure 3A2): the peak value of the actual force that subjects applied after they had been exposed to a vertical series of force-fields (LF = 0.98 ± 0.05(SE) [N]) was similar to the peak value of the actual force when they were exposed to the force-field of *B* = 2.5 Ns/m (mean value of the sequence, LF = 1.07 ± 0.04 [N], blur bar in Figure 3A2) but different from the learned force when they were exposed to the force-field of *B* = 4 Ns/m (the last term of the series, LF = 1.22 ± 0.05 [N]) or *B* = 5 Ns/m (the next term of the series, LF = 1.59 ± 0.07). We also tested the possible implicit learning of the series of force fields during the random-perturbation phase of the experiments. Our findings show clearly that there was no such effect in both experiments. The peak force in each error-clamp trial that followed the series trials was similar (one-way ANOVA found no effect of the series repetition [*F*_(9, 90)_ = 1.16, *P* = 0.33 and *F*_(9, 90)_ = 0.59, *P* = 0.80 for horizontal and vertical experiments, respectively]).

To statistically validate our main observation that subjects predict approximately the average value of the recent force fields, we considered two alternative hypotheses for the expected force-fields in the error-clamp trials that followed the sequence trials and calculated the differences between the actual data and the expected result according to each hypothesis. The first difference—designated “average” difference—was the difference between the value of the actual force in the error-clamp trials when the subjects were exposed to a force-field of *B* = 2.5 Ns/m (in the constant-perturbations phase—blue bar, Figure 3A) and the value of the actual force in the error-clamp trials after the series force-field trials (in the random-perturbations phase—black bar, Figure 3A). The second difference—the “next” difference—was the difference between the value of the actual force in the error-clamp trial when the subjects were exposed to a force-field of *B* = 5 Ns/m (red bar, Figure 3A) and the value of the actual force in the error-clamp trial after the series force-field trials (black bar, Figure 3A). Figure 3B shows that the subjects clearly demonstrated predictive control of the average after performing series of four trials under force fields of increasing magnitudes. A *t*-test was used to assess the difference between each pair of measures. The “average” difference (0.12 ± 0.05 [N] for the horizontal experiment and 0.1 ± 0.06 [N] for the vertical experiment) was significantly lower [two-tailed *t*-test: *t*(99) = 8.51, *P*<0.0001 and *t*(99) = 7.69, *P*<0.0001 for horizontal and vertical experiments, respectively] than the “next” difference [0.69 ± 0.07 (N) for the horizontal experiment and 0.61 ± 0.08 (N) for vertical experiment]. The high positive value of the “next” difference in both experiments clearly supports our main findings that subjects did not predict the future, as was the case in our previous study on a lifting task. The value of the “average” difference indicates that subjects predict a force of lower value than the series average, possibly because in the averaging process they also included in the weighting the prior baseline of the zero force field.

## Discussion

In this study, we monitored goal-directed arm movements with the aim of examining the way in which short-term human motor memory is used to predict force perturbations. Our main finding was that the motor prediction of force perturbations is based on averaging past experience (even after implicitly experiencing four consecutive trials of increasing force perturbations). In contrast to previous studies concentrating solely on horizontal reaching movements, here we also tested vertical reaching movements and found similar results, thereby refuting one possible explanation—that force expectation is based on movement's direction- for the discrepancy between adaptation to force perturbations during reaching movements and adaptation to weight in a lifting task, for which motor memory was demonstrated to predict the next object rather than the average (Mawase and Karniel, 2010).

Our results are consistent with those of previous studies examining the learning of motor tasks in an unpredictable environment (Scheidt et al., 2001; Takahashi et al., 2001; Donchin et al., 2003). Those studies suggested that in a randomly unpredictable environment, motor memory uses a short-term averaging process to learn a motor task and that learning may not represent the statistics of how perturbations change over time. Our findings are also consistent with those of a previous study that examined motor adaptation to predictable perturbations (Thoroughman and Shadmehr, 2000; Karniel and Mussa-Ivaldi, 2002).

In contrast to the above, the present findings are not consistent with our recent findings regarding motor memory of a lifting task (Mawase and Karniel, 2010), in which we observed that grip force is correctly adjusted to the next load in a simple implicit linear series with increasing weights, thus predicting the future rather than exploiting the past. Predicting the future rather than using the average of the past was also shown in the study of Witney et al. (2001), which clearly found that the predictive response of the grip force in future trials is proportional to the predictions of the last three trials. Taken together, the results of previous studies on grasping and those of the current study on reaching suggest that the brain can generate at least two different types of motor representation, one exploiting the past for reaching movements and the other predicting the future for grasping movements. Initially, we hypothesized that the difference between the tasks might be due to the effect of gravity and the fact that lifting is performed vertically while reaching may be performed horizontally or vertically. This hypothesis is clearly refuted by the current study, in which we found that adaptation to force perturbations in vertical reaching is similar to adaptation during horizontal reaching. We therefore examined three other possibilities—discussed below—that could explain the apparent difference in predictions between force-field and weight adaptation.

**Figure 4:**
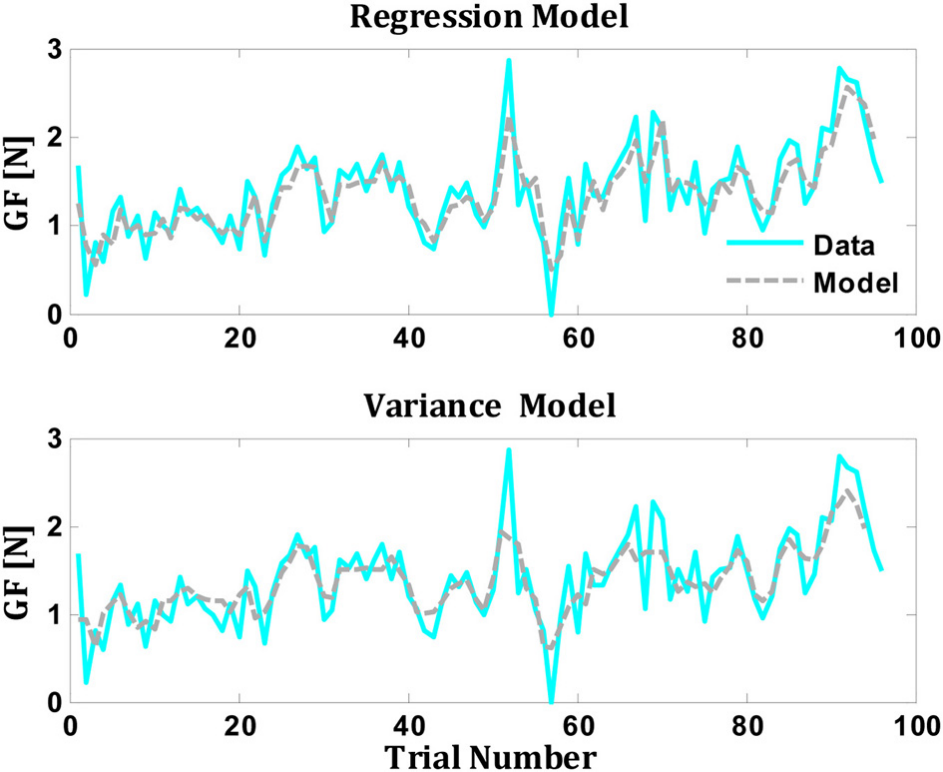
**Comparison between predictions of the regression and variance models for grip force from single subject (Data from Mawase and Karniel, 2010)**. The gray line represents the actual performance of the subject, while the colored line represents the model's prediction (upper panel for regression model and the lower panel for the variance model).

1. *Perturbation familiarity*. The weight of an object represents a very familiar load that can easily be mapped to real objects. It was suggested that the motor memory is very accurate in representations familiar weights with broad range of physical properties (Gordon et al., 1993); thus, implicitly the use of representations from motor memory, including extrapolation, would be highly possible. Conversely, curl field viscosity represents an unusual novel load that cannot be easily mapped to external objects and averaging over unusual perturbations rather than extrapolation might be a more plausible scenario for reaching tasks.
2. *Environmental variability*. We found that in reaching tasks the lateral force was significantly robust to the environmental variability (force field amplitudes) and that subjects based their predictions on the average of the previous trials (Figure 3). In lifting tasks, two mechanisms have been proposed to account for the observations of higher grip force when we lift series of increasing objects: in the first, it is posited that grip force might change with the environmental variability (object weights) along with applying safety margin to avoid slipping of the object (Hadjiosif and Smith, 2011). Second, grip force might change according to the prediction of the next weight based on linear regression of the previous three trials (Mawase and Karniel, 2010). To investigate the two different possibilities in a lifting task, we used two different trial-by-trial adaptation models, since models of this type had previously been shown to be extremely successful in modeling motor adaptation (Thoroughman and Shadmehr, 2000; Scheidt et al., 2001; Donchin et al., 2003). The first model, the regression model, evaluated the dependence of the grip force on the average grip force of previous trials and the predicted force based on linear regression of the previous grip forces. This model suggested that subject prediction in each trial is a linear combination of the average of the previous grip forces and the predicted forces based on regression of the previous grip forces (Mawase and Karniel, 2010). The second model, the variance model, evaluated the dependence of the grip force on the average grip forces of previous trials and the variance of the previous weights.
  ***Regression model:***
  (3) $$\hat{G}F[n]=w_{1}\;\cdot \;\mu [n]+w_{2}\;\cdot \;\hat{G}F_{rgress}[n],\;\{\begin{array}{c} \mu [n]=mean(GF[n-3:\;n-1])\\ \hat{G}F_{rgress}[n]=regression(GF[n-3:\;n-1]) \end{array}$$
  where $\hat{G}F[n]$ is the predictive grip force in trial *n*, μ[*n*] is the average of the previous three grip forces and $\hat{G}F_{rgress}\left[n\right]$ is the linear regression estimation of the previous three grip forces.
  ***Variance model:***
  (4) $$\hat{G}F[n]\;=w_{1}\;\cdot \;\mu \left[n\right]+w_{2}\;\cdot \;M_{std}[n],\;\{\begin{array}{c} \mu [n]=mean(GF[n-3:\;n-1])\\ M_{std}[n]=std(Weight[n-3:\;n-1]) \end{array}$$
  where *M*_*std*_[*n*] is the standard deviation of the previous three weights. It is important to note that the two models contain two adjustable parameters (*w*_1_ and *w*_2_) and therefore they can be compared without any corrections for differences in complexity or any risk of over fitting. We have fitted the two parameters of each model to each subject. The performance of the models was quantified by measuring the correlation and the VAF between the data and the model's prediction. We found that both models fitted well the data (Figure 4). The mean percentage of VAF in the regression model was 0.60 ± 0.02 (SE) across all subjects, with a correlation coefficient of 0.77 ± 0.01 (model coefficients were on average *w*_1_ = 0.66 ± 0.03 and *w*_2_ = 0.32 ± 0.03). The mean percentage of VAF for the variance model was 0.56 ± 0.05 (SE) across all subjects, with a correlation coefficient of 0.75 ± 0.04 (model coefficients were on average *w*_1_ = 0.81 ± 0.03 and *w*_2_ = 0.25 ± 0.02). Further studies are required to decipher these two interpretations.
3. *Brain areas*. Our results support the idea that motor behavior in reaching and grasping is processed independently and might rely on different anatomical locations in the brain (Bracha et al., 2000; Diedrichsen et al., 2005; Krakauer and Shadmehr, 2006). Recent studies on human neuroimaging and monkey neurophysiology have suggested that different brain neural circuits in the posterior parietal cortex are directly involved in the above two motor tasks, with the superior parietal to dorsal premotor cortex circuit being involved in reaching movements and the inferior parietal and inferior frontal circuit being more often involved in grasping (Rizzolatti and Matelli, 2003; Tunik et al., 2005; Creem-Regehr, 2009). Additionally, a growing number of human neuroimaging studies are providing insight into the involvement of the medial intraparietal area (MIP) in reaching movements and of the anterior intraparietal area (AIP) in grasping movements (Karnath and Perenin, 2005; Davare et al., 2007, 2010, 2011; Grafton, 2010).

In summary, the reported study explored the use of motor memory for predicting force fields during reaching movements. We clearly demonstrated that in adaptation to force perturbations the prediction is based on the average of past experience rather than on extrapolation to the future, as had previously been demonstrated in grasping and lifting as well as in the recent literature regarding episodic memory. Further study is required to elucidate the role of motor memory in motor learning and the similarities and differences between the various types of neural structures responsible for learning and memory.

### Conflict of interest statement

The authors declare that the research was conducted in the absence of any commercial or financial relationships that could be construed as a potential conflict of interest.
